# The role of “red flags” in the diagnostic work-up of hereditary transthyretin amyloidosis: a study using a machine-learning approach

**DOI:** 10.1007/s10072-026-09285-w

**Published:** 2026-08-12

**Authors:** Antonia Pignolo, Giada Sajeva, Christian Messina, Maria Magro, Nicasio Rini, Paolo Alonge, Gabriele Gerbino, Manfredi Parasporo, Vincenzo Di Stefano, Filippo Brighina

**Affiliations:** 1 Department of Biomedicine, Neuroscience and Advanced Diagnostics (BiND), University of Palermo, Via del Vespro 143, Palermo, 90127 Italy; 2 Demetrix s.r.l., Palermo, 90146 Italy

**Keywords:** Transthyretin amyloidosis, ATTRv, Red flags, Machine learning, Explainable artificial intelligence, Genetic screening

## Abstract

**Introduction:**

Hereditary transthyretin amyloidosis (ATTRv) is a rare progressive, potentially life-threatening multisystem disorder caused by mutations in the transthyretin (TTR) gene, with variable penetrance and heterogeneous phenotypes, often leading to diagnostic delays, particularly in non-endemic regions. Identifying clinical “*red flags*” is crucial to shorten diagnostic latency. Machine learning (ML), a branch of artificial intelligence (AI), together with explainable artificial intelligence (XAI), offers novel opportunities to refine diagnostic algorithms and prioritize predictive features in ATTRv.

**Materials and methods:**

A total of 452 patients who underwent TTR genetic testing between 2019 and 2024 in Sicily were retrospectively analyzed. Genetic testing was performed via polymerase chain reaction (PCR) and sequencing of TTR exons 2–4. Patients were stratified into Western and Eastern Sicily sub-cohorts to train and validate supervised ML models. Multiple algorithms were compared with hyperparameter tuning via GridSearch with cross-validation. Model interpretability was ensured using SHapley Additive exPlanations (SHAP) values and permutation importance.

**Results:**

Among 452 patients, 68 (15%) carried a TTR mutation, 51.5% of whom were symptomatic. The most frequent red flags were sensory neuropathy (62.6%) and family history of cardiomyopathy (51.8%). Tree-based models outperformed other algorithms, with Random Forest selected for its optimal balance between precision and recall. Bilateral carpal tunnel syndrome, family history of neuropathy, and ataxia emerged as the most informative predictors.

**Discussion:**

These findings suggest that integrating ML with clinical red flags may support the diagnostic decision-making process in patients referred for suspected ATTRv. However, these results should be considered exploratory and require validation in independent cohorts before clinical implementation.

## Introduction

Hereditary transthyretin (TTR) amyloidosis (ATTRv) is a rare disorder characterized by a wide spectrum of clinical manifestations, which may present with predominantly neurological, predominantly cardiac, or mixed phenotypes [1]. More than 150 mutations in the TTR gene have been identified in ATTRv amyloidosis [1]. Most pathogenic variants are missense point mutations inherited in an autosomal dominant manner, with highly variable penetrance and phenotypic expressivity, even among members of the same family [1]. The phenotypic expression of pathogenic TTR mutations varies in terms of cardiac and neurological involvement, age of onset, and disease progression [1, 2]. While most known mutations are associated with a mixed phenotype affecting both the peripheral nerves and the heart, substantial variability can be observed even among individuals carrying the same mutation, primarily due to incomplete penetrance [1, 3, 4]. In regions where ATTRv amyloidosis is endemic, the diagnosis is typically made within one year of symptom onset [5]. However, in non-endemic areas, diagnostic delays of 3–4 years are common [5]. This is often due to factors such as the absence of a known family history and the wide range of initial clinical manifestations, which can mimic other peripheral neuropathies [5]. Early diagnosis of ATTRv amyloidosis remains challenging, prompting several studies aimed at identifying clinical red flags to support earlier recognition of the disease [5]. According to Conceição et al., ATTRv amyloidosis should be suspected in patients presenting with progressive peripheral sensory-motor neuropathy accompanied by one or more of the following features: family history of neuropathy, autonomic dysfunction, cardiac hypertrophy, gastrointestinal disturbances, unexplained weight loss, carpal tunnel syndrome, renal impairment, or ocular involvement [5, 6]. Artificial intelligence (AI) is a branch of computer science dedicated to developing software capable of performing tasks that emulate human intelligence [7]. AI encompasses a wide spectrum of computational systems designed to replicate cognitive functions such as problem-solving, reasoning, pattern recognition, and knowledge acquisition [7]. Among the most commonly applied forms of AI in healthcare are machine learning (ML) and natural language processing, both of which allow for in-depth analysis of complex datasets to uncover previously unrecognized patterns and associations [7]. Although distinct, these two approaches are interconnected, with natural language processing often relying on ML to extract meaning from language [7]. The capabilities of ML hold promise for enhancing diagnostic accuracy, guiding the development of new therapies, and deepening the understanding of disease progression [7]. In recent years, ML has been increasingly applied with promising results across various domains of medical research, including disease diagnosis from neuroimaging, classification and prediction of disease progression, gene regulatory network analysis, gene prioritization, and disease subtype classification [7]. For instance, in the field of neurology, ML has been employed both for diagnostic purposes and to assess disease progression in a variety of conditions, including dementias, mild cognitive impairment, Parkinson’s disease, multiple sclerosis, amyotrophic lateral sclerosis (ALS), vertigo, and epilepsy [7–10]. Recently, an AI-driven framework for the automatic detection of red flag symptoms related to ATTRv within electronic health records has been validated; additionally, ML has shown potential as a valuable tool to identify patients with neuropathy who may benefit from genetic testing for ATTRv [11, 12]. This study aimed to evaluate the role of ML in identifying the most clinically relevant red flags associated with TTR mutation positivity, and to assess its ability to support the diagnostic decision-making process in a real-world cohort of patients referred for suspected ATTRv, including both symptomatic individuals undergoing diagnostic workup and asymptomatic subjects referred due to a confirmed family history of TTR mutation.

## Materials and methods

The study was approved by the Ethical Committee of Palermo on 13 July 2020 (V. n.7/2020) and was conducted in conformity with the Declaration of Helsinki principles.

Clinical variables, including predefined red flags, were extracted from medical records and independently reviewed by experienced clinicians. In particular, data collection was performed by neurologists with expertise in the diagnosis and management of ATTRv. When available, operational definitions were applied to ensure consistency in data collection, and data harmonization across centers was performed to minimize variability in variable coding. Bilateral Carpal Tunnel Syndrome (BCTS) was defined as electrophysiologically confirmed involvement of both wrists. Autonomic dysfunction was defined based on documented symptoms (e.g., orthostatic hypotension, gastrointestinal or urinary dysfunction), results of autonomic testing when available, and/or standardized questionnaire scores (e.g., Composite Autonomic Dysfunction Test, CADT), when applicable.

Patients were recruited in a tertiary referral center for amyloidosis. Clinical evaluation and data collection were primarily conducted from a neurological perspective, as most patients were referred for neurological suspicion of ATTRv. Patients suspected to have ATTRv based on specific “red flags” were enrolled. In a second phase, they underwent a comprehensive diagnostic workup for ATTRv, including genetic testing. Clinical data have been retrospectively collected from patients undergoing *TTR* genotyping. For each patient undergoing genetic testing, the presence of specific “red flags” was investigated. Then, clinical data were compared in patients with positive and negative genetics; afterwards, the red flags included in this study were combined to define a precise algorithm for diagnosis through a “Machine Learning” model.

### Patients’ population

In this study, patients who underwent genetic testing for *TTR* mutations were included. A Genilam swab test was performed for each patient. This test employed Polymerase Chain Reaction (PCR) targeting exons 2, 3, and 4 of the TTR gene (NM_000371.4), followed by automated sequencing using the SeqStudio Flex Genetic Analyzer. This method enabled the detection of small point mutations, deletions, or insertions (< 20 bp) in the specified gene regions. The genetic analyses included in this study were conducted between June 2019 and August 2024.

The study cohort included both symptomatic patients undergoing diagnostic workup for suspected ATTRv and asymptomatic individuals referred for genetic testing due to a confirmed family history of TTR mutation. Demographic data were collected for each patient who underwent genetic testing for *TTR* gene mutations, including age, sex, and the presence or absence of a *TTR* mutation (along with the specific mutation, if present).

**Fig. 1:**
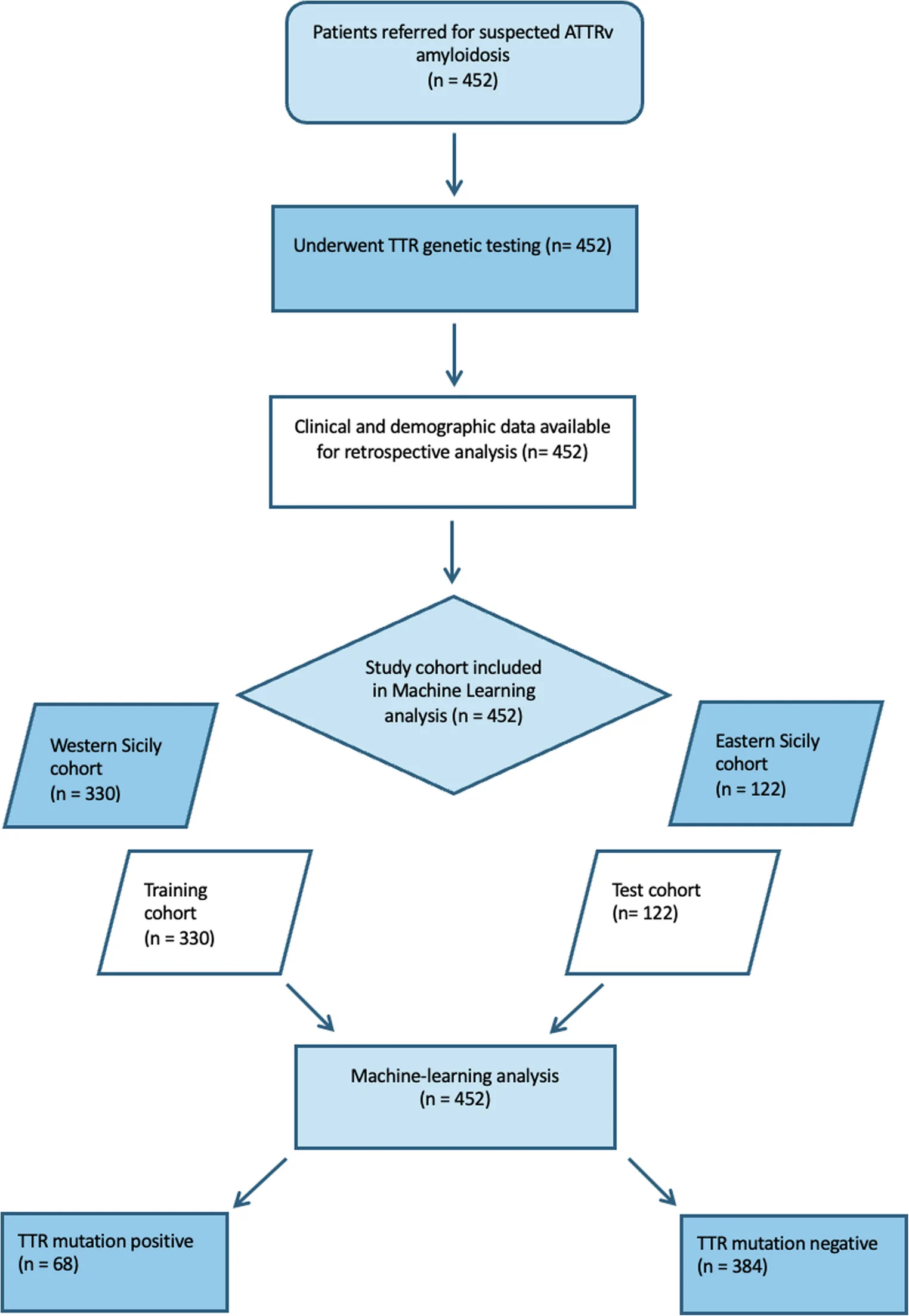
Flowchart of patient selection and study design. Consecutive patients referred for suspected hereditary transthyretin amyloidosis (ATTRv) between June 2019 and August 2024 and undergoing TTR genetic testing were screened for inclusion. All eligible patients with available clinical and demographic data were included in the retrospective analysis. The study cohort was subsequently divided into Western Sicily and Eastern Sicily sub-cohorts for model development and validation. Overall, 68 patients were found to carry a pathogenic TTR variant, whereas 384 tested negative

Additionally, past medical history was gathered, including the presence of autoimmune diseases, prior diagnoses of chronic inflammatory demyelinating polyneuropathy (CIDP) or ALS, diabetes, and any previous misdiagnoses. Clinical data were recorded, such as the presence or absence of symptoms. Furthermore, red flags were systematically documented, including unexplained weight loss, BCTS, sensory neuropathy, lumbar canal stenosis, ataxia, gastrointestinal disturbances, autonomic dysfunction, cardiomyopathy, positive bone or cardiac scintigraphy, renal dysfunction, vitreous opacities, positive salivary gland biopsy, family history of cardiomyopathy, family history of neuropathy, and family history of ATTRv. Each item from the past medical history, clinical data, and red flag checklist was encoded in a binary format, with ‘0’ indicating absence and ‘1’ indicating presence. Variables related to instrumental investigations, such as bone scintigraphy, cardiac uptake, biopsy, and positive biopsy, were recorded as paired variables: one indicating whether the examination was performed (bone scintigraphy and biopsy), and one indicating whether the result was positive (cardiac uptake and positive biopsy), reflecting the logical dependency between these variables in the diagnostic pathway. All available data, including demographic information, medical history, clinical features, and red flags, were systematically collected and integrated into a structured dataset for subsequent analysis. Then, the total cohort was divided into two subcohorts: the Western Sicily subcohort, including patients from Palermo, Trapani, and Agrigento, and the Eastern Sicily subcohort, comprising patients from Catania, Enna, and Siracusa (Fig. 1).

Eligibility criteria included: age ≥ 18 years, provision of informed consent for genetic testing, and the presence of at least two clinical red flags suggestive of ATTRv amyloidosis. Red flags were systematically assessed through a standardized questionnaire administered during outpatient evaluation at each participating center. Exclusion criteria were: lack of informed consent, age < 18 years, known haematological disorders potentially associated with secondary amyloidosis, established secondary amyloidosis, and non-eligibility for genetic testing. For cascade screening, genetic testing was offered according to expert consensus recommendations and current guidelines, taking into account the predicted age of disease onset (PADO).

### Algorithmic modelling and feature attribution analysis

To investigate which red flags are most strongly associated with the pathogenic gene mutation, explainable artificial intelligence (XAI) techniques were employed. After an initial data preprocessing phase, several algorithms were implemented, including both linear and non-linear models suitable for categorical variables. Multiple GridSearch cycles were performed using different feature combinations. The variable *family history of ATTRv* was excluded, given the autosomal dominant inheritance of the disease, while age was not considered, as it only reflects the time of diagnosis. For each model, hyperparameter tuning was conducted based on the available dataset and the red flag variables. Once the evaluation metrics were computed for each model, selected XAI methods were applied. These techniques enable the interpretation and understanding of ML model behaviour, allowing the explanation of predictions and the identification of the decision-making processes underlying the models.

### Model training

The selected classification models are based on supervised learning algorithms, which require a labelled dataset for training. These models operate as binary classifiers, in line with the binary structure of the output variable. The training and evaluation pipeline included the following phases:

1. **Data Preprocessing**. Data cleaning and preprocessing were performed based on the insights derived from the previous exploratory data analysis. This step ensured the removal of inconsistencies and the transformation of variables into appropriate formats for model training.
2. **Feature Selection**. In the initial training phase, all variables in the dataset were considered except for *age* and *family history of ATTR*, which were excluded due to their limited informative value or potential redundancy. Variable family history of ATTRv was excluded because of the autosomal dominant inheritance of the disease, while Age was excluded since it represents age at the time of genetic testing rather than age of symptom onset. Given that, the cohort includes both symptomatic patients undergoing diagnostic workup and asymptomatic individuals referred for genetic testing due to a confirmed family history of TTR mutation, age at genetic testing primarily reflects the reason for referral rather than the biological characteristics of the disease, representing a potential confounding variable rather than a diagnostically informative predictor [1, 5]. Feature selection was then performed iteratively to assess the impact of specific predictors related to a family history of neurological and/or cardiovascular disorders. In addition, the variables *bone scintigraphy* and *biopsy of salivary gland*, were also considered for their role as supporting features for the diagnostic outcome. In fact, these variables act as logical prerequisites for the results of *cardiac uptake* and biopsy variables. Various subsets of red flags were systematically assessed by iteratively excluding features that were potentially confounding or less informative.
3. **Train-Test Split**. The dataset was split into a training set and a test set. Specifically, data from facilities located in Western Sicily were used for training, while data from Eastern Sicily were used for validation. The Eastern Sicily sub-cohort was kept entirely aside from the outset and was never used in any phase of model development, preprocessing, or feature selection. This design constitutes a geographically stratified evaluation on an independent dataset, which goes beyond a simple internal validation as defined by TRIPOD + AI. However, as both sub-cohorts share a similar regional genetic background and red-flag ascertainment context, generalizability beyond the Sicilian setting should be interpreted with caution.
4. **Hyperparameter Tuning**. Hyperparameter optimization was performed through a two-phase strategy using GridSearchCV with Repeated Stratified 10-fold Cross-Validation (20 repeats). In the preliminary screening phase, a wide range of algorithms from different families were compared, including Logistic Regression, SVC, K-Nearest Neighbours, Naive Bayes, Random Forest, Gradient Boosting, XGBoost, and MLP. Tree-based models and their ensemble variants consistently outperformed the others across different feature combinations. The second phase focused on an in-depth evaluation of decision tree–based algorithms (Random Forest, ExtraTrees, Gradient Boosting, XGBoost, LightGBM, CatBoost, and Decision Tree), benchmarked against linear classifiers. Hyperparameters were tuned to maximize the F1-score, chosen for its robustness under class imbalance. This rigorous validation strategy mitigated biases from random data partitioning, improved reproducibility, and ensured that model selection relied on a multi-objective assessment rather than a single performance metric.
5. **Model Evaluation**. After training, model performance was evaluated on the test set. Evaluation metrics included accuracy, precision, recall, F1-score, and the confusion matrix, allowing for a comprehensive assessment of classification performance. To evaluate both the stability and reliability of the reported performance estimates, 95% confidence intervals were computed for all metrics using a bootstrap resampling approach on the test set. A total of 1000 bootstrap iterations were performed by resampling the test set with replacement; for each iteration, all metrics were recomputed, and the 95% confidence intervals were derived from the 2.5th and 97.5th percentiles of the resulting distributions. The goal was to simultaneously maximize the detection of positive cases (evaluating Recall), ensure model robustness and promote model generalization. A further goal was to go beyond a simple performance evaluation to explore the model’s own decision-making process, using global **Explainable AI (XAI)** methods [13].

### Permutation importance

**Fig. 2:**
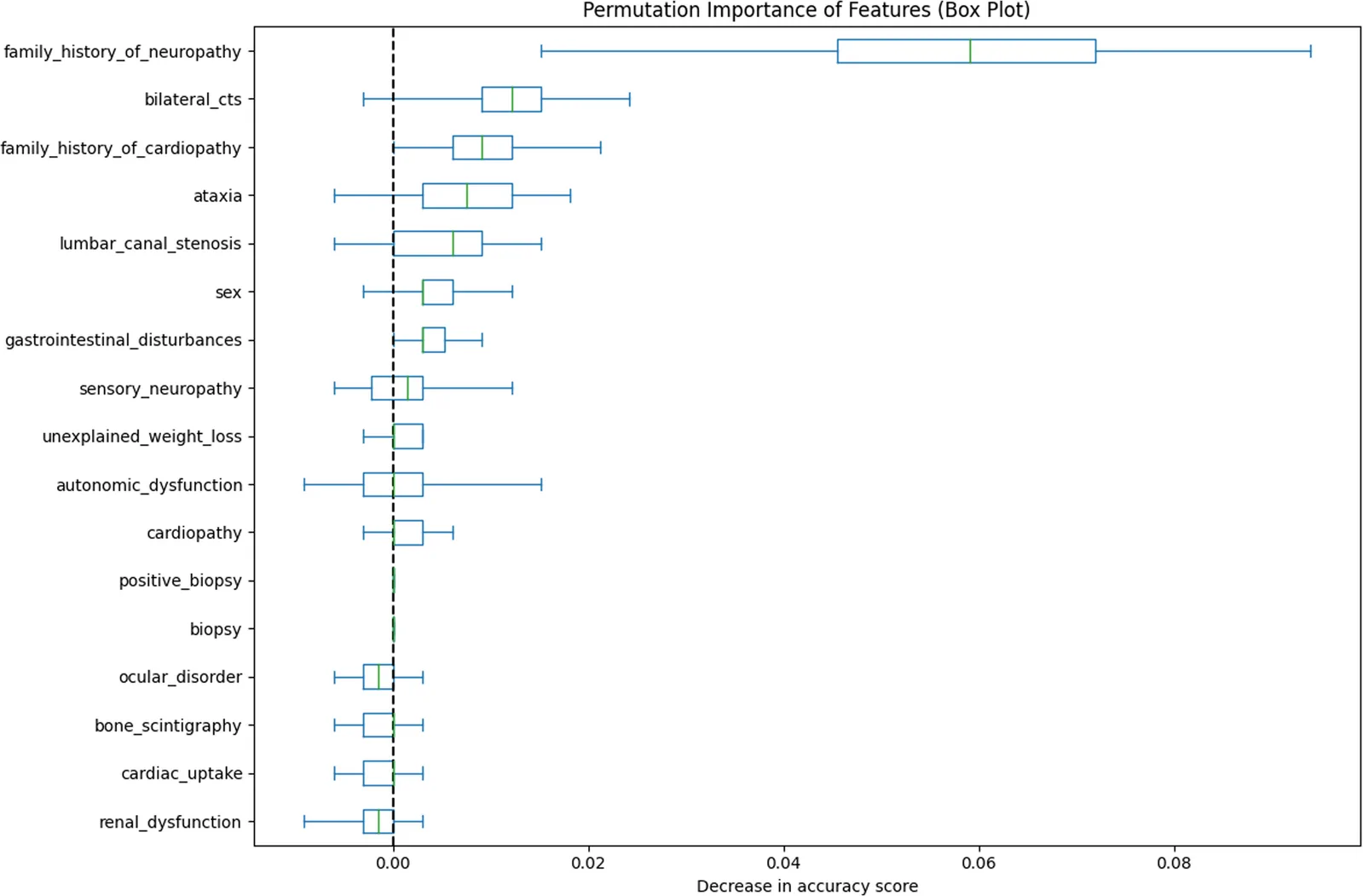
Box plot showing the permutation importance of features in the predictive model. The x-axis represents the decrease in accuracy score after permutation of each feature. Higher values indicate greater importance for the model performance. Abbreviations: CTS, carpal tunnel syndrome

Permutation Importance is a model-agnostic technique used to assess the relevance of input features in a predictive model (Fig. 2). This approach can be applied to any machine learning algorithm and offers an intuitive way to interpret the contribution of individual variables to the model’s predictions. The procedure involves the following steps:

1. **Baseline Performance Calculation**: The model’s predictive performance is first evaluated using the original test dataset.
2. **Feature Permutation**: For each feature, its values are randomly shuffled across the test set, effectively disrupting the relationship between that feature and the target variable.
3. **Performance Recalculation**: The model’s performance is then re-evaluated using the permuted dataset.
4. **Importance Estimation**: The importance of each feature is determined by computing the difference between the original and the permuted performance scores. A substantial drop in performance suggests that the feature plays a crucial role in the model’s predictions

### SHAP (SHapley additive exPlanations)

**Fig. 3:**
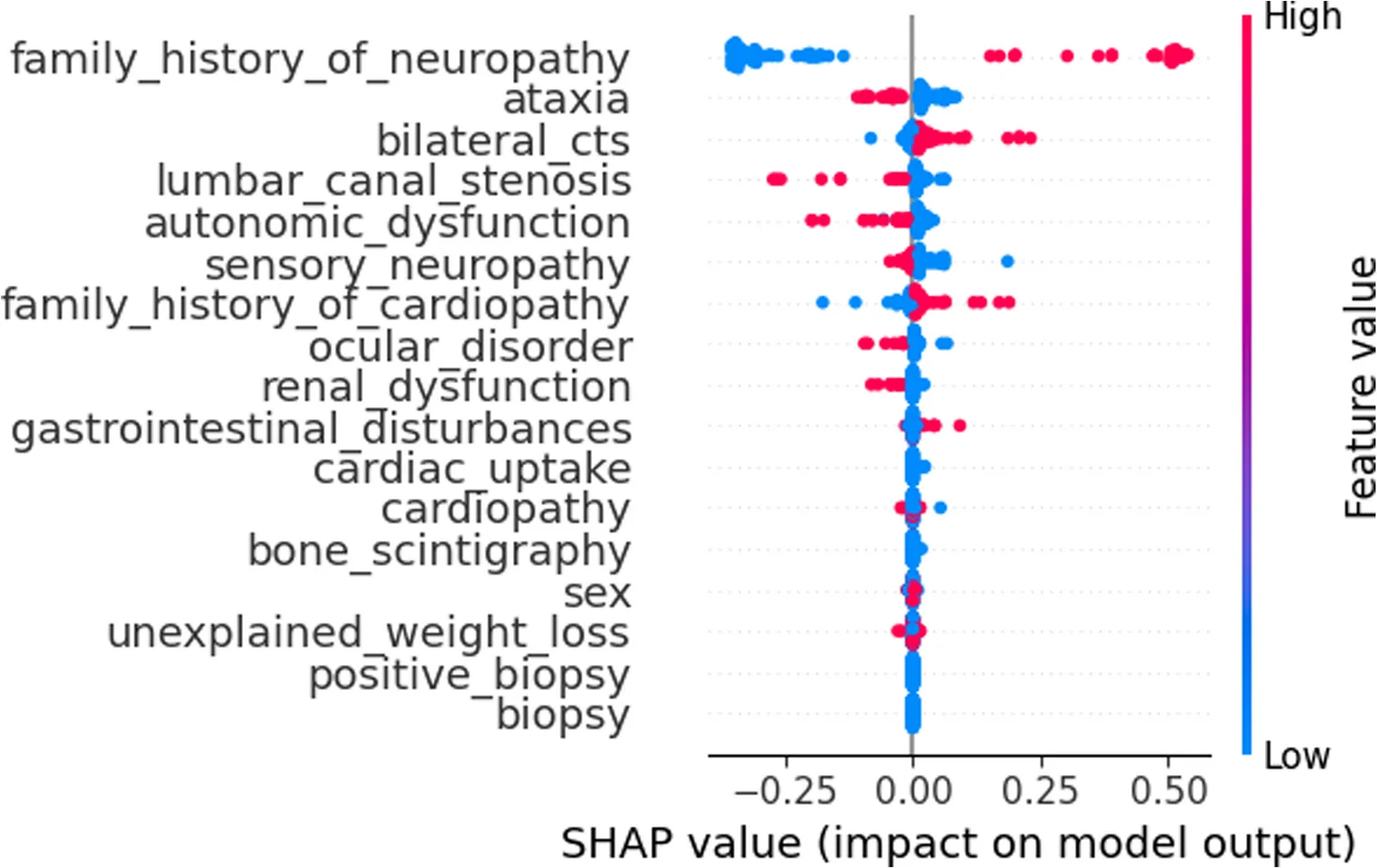
SHAP summary plot showing the impact of each feature on the model output. Each dot represents an individual observation. The x-axis shows the SHAP value (impact on model prediction). Red dots indicate the presence of the red flag (higher feature value), while blue dots indicate its absence (lower feature value). Points on the right side of the plot contribute to the prediction of a TTR mutation, whereas points on the left contribute to the prediction of no TTR mutation. Abbreviations: CTS, tunnel carpal syndrome

SHAP is an interpretability technique for predictive models based on cooperative game theory. It assigns each feature a value (Shapley value) that quantifies the average marginal contribution of that feature to the model’s prediction, considering all possible feature combinations (Fig. 3). SHAP quantifies the contribution of each feature to the prediction by calculating how much the prediction changes when the feature is included versus when it is excluded, considering all possible combinations of features. This approach, grounded in game theory, ensures that the attribution of importance is both fair and consistent across all features. The individual contributions, called Shapley values, add up to the final prediction, allowing a clear decomposition of the model’s output. This makes it possible to understand not only the overall influence of each feature across the entire dataset but also how each feature impacts specific individual predictions. This dual capability makes SHAP especially useful in clinical settings where understanding both the global behaviour and local decision-making of the model is essential.

### Statistical analysis

**Fig. 4:**
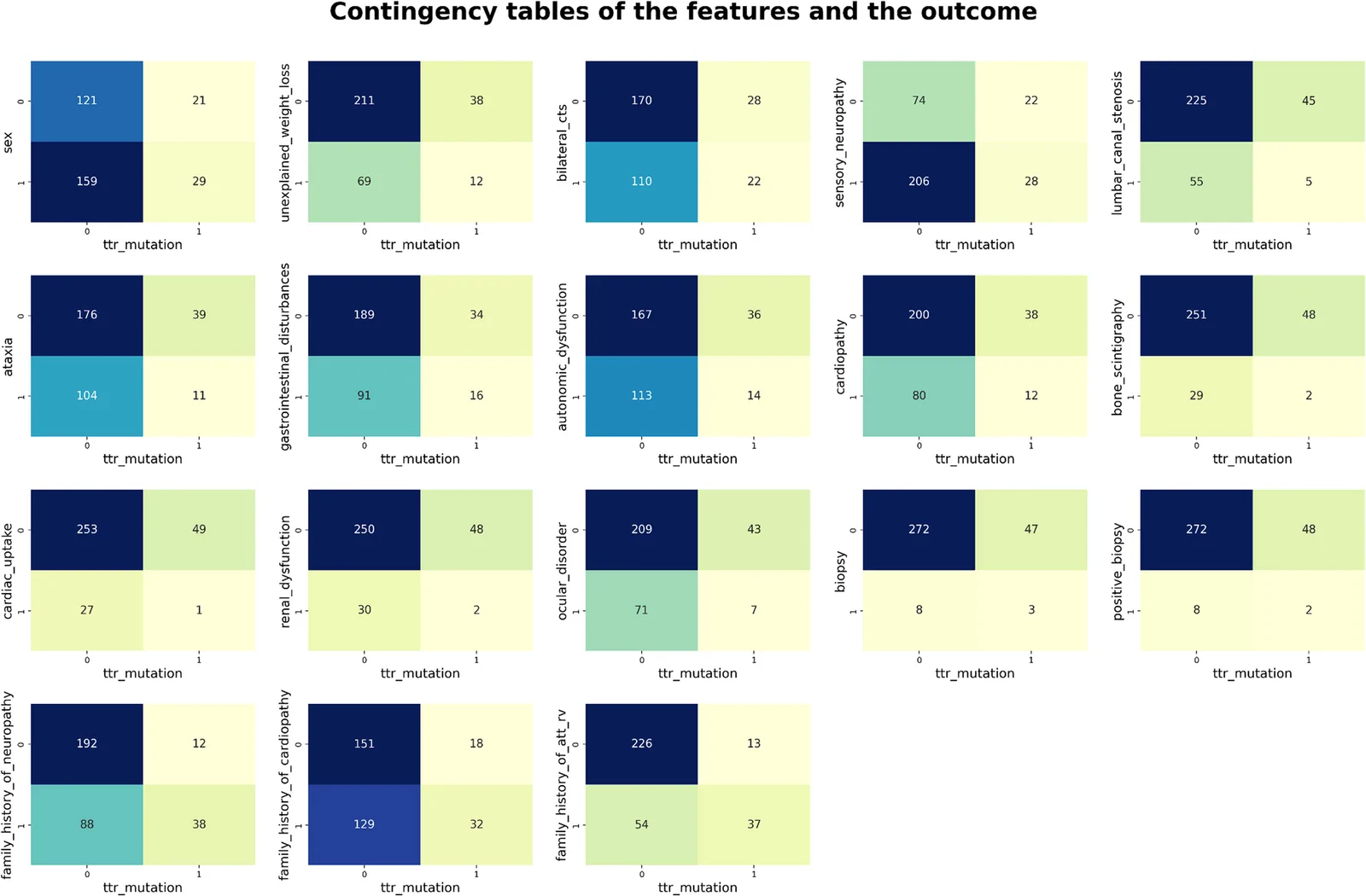
Contingency tables showing the distribution of each clinical feature according to the outcome (TTR mutation status). Each heatmap represents the number of patients with or without the specific feature stratified by TTR mutation status (0 = no mutation, 1 = mutation). Color intensity reflects the frequency of observations within each cell. Abbreviations: CTS, carpal tunnel syndrome

All clinical variables included in the analysis were fully available, with no missing data, owing to systematic data collection across the participating referral centers. Age is presented as both a continuous variable (mean, standard deviation, and range) and in raw values, while categorical variables are presented in raw numbers and percentages. The chi-square test was performed between each input variable and the target outcome exclusively on the Western Sicily sub-cohort (training set), to assess the statistical association between individual features and the outcome. The chi-square results are reported in Table 1. This analysis was not used as a formal feature selection criterion, but it was intended as a complementary statistical tool to evaluate the consistency between traditional univariate associations and the results obtained via explainable AI methods (SHAP values and permutation importance). Feature selection was conducted through an iterative analytical process combining clinical reasoning and repeated GridSearch cycles with different feature combinations, as described in the Algorithmic Modeling and Feature Attribution Analysis section. Box plots and bar charts were employed to visually depict the relative importance of the identified red flags (Fig. 4).

**Table 1:** Chi-squared and p-value test results. The table reports the Chi-squared statistics (${X}^{2}$) and the corresponding exact p-value for each evaluated feature, quantifying the strength of the association with the outcome. Features are ranked in descending order of statistical significance

Feature	Chi-squared	*p*-value
sex	0.0	1.0
positive_biopsy	0.0037272973705611717	0.9513181180146255
bilateral_cts	0.16827124397902254	0.6816528000433049
unexplained_weight_loss	0.17080318206316047	0.6793989786992809
lumbar_canal_stenosis	0.1778891701741033	0.6731935650577011
gastrointestinal_disturbances	0.23481973434535106	0.6279728702009639
cardiopathy	0.678609705530957	0.41006587845291986
cardiac_uptake	0.9972618613777491	0.3179739655690401
renal_dysfunction	1.1462843898356911	0.2843283098408752
ataxia	1.8648396114373864	0.17206727730126814
ocular_disorder	2.9111556892491084	0.08796881709419216
family_history_of_cardiopathy	2.943998427461299	0.0861971182687599
autonomic_dysfunction	4.01895603968309	0.04499155491335869
sensory_neuropathy	7.010211228435379	0.00810461174666056
family_history_of_neuropathy	34.59868813037612	4.051792591161303e-09

## Results

**Fig. 5:**
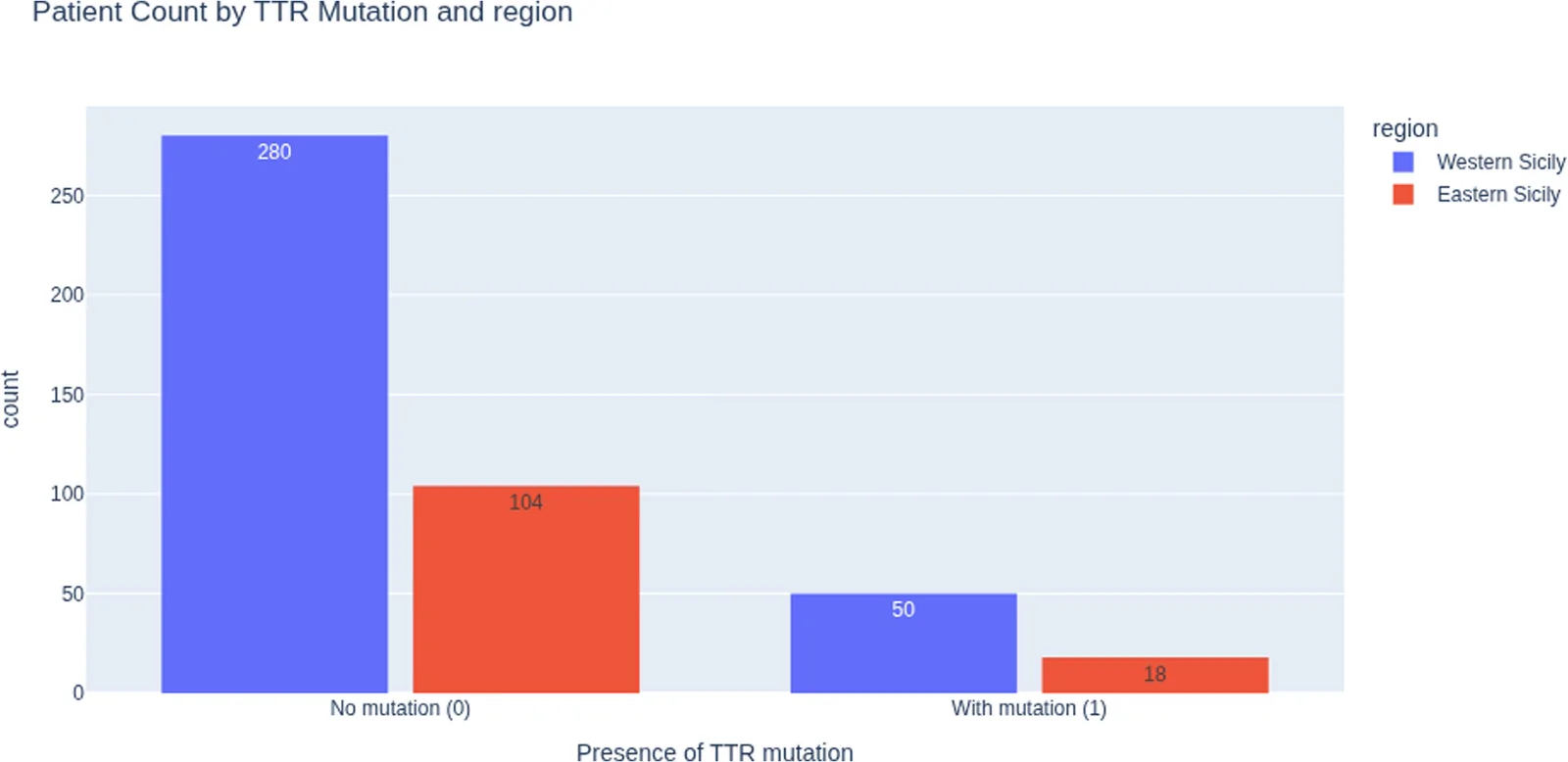
Patient distribution according to TTR mutation status in Western and Eastern Sicily. Bars represent the number of patients without TTR mutation (0) and with TTR mutation (1) in each region. Abbreviations: TTR, transthyretin

In total, data were collected from 452 patients, including 210 males (46.5%) and 242 females (53.5%). The mean age was 61 ± 15 years (range 19–86 years). In total, 68 patients (15.0%) were found to carry a mutation in the *TTR* gene in Western and Eastern Sicily (Fig. 5); among these, 35 individuals (51.5%) were symptomatic. Among the 452 patients tested for *TTR* mutations, the most frequently observed red flag was sensory neuropathy, present in 283 cases (62.6%), as shown in Table 1. A family history of cardiomyopathy was reported in 234 patients (51.8%), while bilateral carpal tunnel syndrome was found in 209 cases (46.2%). Both a family history of neuropathy and autonomic dysfunction were noted in 166 patients (36.7%), gastrointestinal symptoms in 140 patients (31.0%), and ataxia in 142 patients (31.4%). Unexplained weight loss was reported in 152 individuals (33.6%), and a family history of ATTRv in 123 (27.2%). Cardiac involvement was observed in 116 patients (25.7%), lumbar spinal stenosis in 78 patients (17.3%), and vitreous opacities in 88 patients (19.5%). Less frequently, renal dysfunction was found in 45 cases (10.0%), positive bone scintigraphy in 31 (6.9%), positive cardiac scintigraphy in 28 (6.2%), and positive salivary gland biopsy in 10 patients (2.2%). In total, 16 patients (3.5%) received a misdiagnosis. Among the misdiagnosed cases, the erroneous diagnoses were as follows: 5 cases of ALS (31.3%), 3 cases of CIDP (18.8%), 3 cases of anorexia nervosa (18.8%), 2 cases of chronic idiopathic axonal polyneuropathy (CIAP) (12.5%), 1 case of diabetic polyneuropathy (6.3%), 1 case of lumbar spinal stenosis (6.3%), and 1 case of dilated cardiomyopathy (6.3%). CIDP misdiagnoses were made based on clinical and electrophysiological findings consistent with the 2021 EAN/PNS diagnostic criteria at the time of evaluation. The case of lumbar spinal stenosis represented an isolated structural diagnosis without electrophysiological evidence of concomitant polyneuropathy on nerve conduction studies.

Out of the total 452 patients, 330 (72.9%) belonged to the Western Sicily cohort, while 122 (27.1%) were part of the Eastern Sicily cohort. All included patients fulfilled the predefined eligibility criteria, including the presence of at least two red flags suggestive of ATTRv amyloidosis.

The chi-square analysis performed on the training set revealed a statistically significant association with TTR mutation status for family_history_of_neuropathy (χ²=34.599, *p* < 0.001), sensory_neuropathy (χ²=7.010, *p* = 0.008), and autonomic_dysfunction (χ²=4.019, *p* = 0.045). Notably, bilateral CTS showed a non-significant univariate association (χ²=0.168, *p* = 0.682), yet emerged as the second most important predictor in the permutation importance analysis. These results indicate the ability of tree-based ML models to capture non-linear interactions that univariate statistical tests cannot detect [14].

A comprehensive analysis of machine learning models confirmed that the best feature combinations included *biopsy*, *bone scintigraphy*, *family history of neuropathy*, and *family history of cardiopathy*. While a simpler configuration without *biopsy* and *bone scintigraphy* also yielded satisfactory results, their retention is justified on grounds of real-world clinical context. Furthermore, comparative analysis of trained models confirmed the superiority of **tree-based algorithms**—specifically **Random Forest** and **XGBoost**—which outperformed the other models considered in this study. In particular, Random Forest model was selected among XGBoost as the final model due to its optimal balance of performance and clinical reliability. Despite XGBoost achieved a perfect Recall of 1.0000 (1.0000, 1.0000), its Precision of 0.3961 (0.2449, 0.5455) showed greater uncertainty, Random Forest’s F1-score of 0.5810 (0.4091, 0.7273) and ROC-AUC of 0.8359 (0.7625, 0.8968) reflect a more balanced and stable performance profile, better addressing the clinical need to minimize false positives and the associated risk of overdiagnosis (Table 2). This, combined with its robust generalization, makes Random Forest the most solid and balanced solution for the task (Table 3).

**Table 2:** Distribution of clinical features according to TTR mutation status in the overall cohort and by geographic area (Western and Eastern Sicily). Data are presented as n (%)

Feature	Total sample (*N* = 452)	Western Sicily (*N* = 330)	Eastern Sicily (*N* = 122)
ataxia	142 (31.42%)	115 (34.85%)	27 (22.13%)
autonomic_dysfunction	166 (36.73%)	127 (38.48%)	39 (31.97%)
bilateral_CTS	209 (46.24%)	132 (40.00%)	77 (63.11%)
biopsy	11 (2.43%)	11 (3.33%)	0 (0.00%)
bone_scintigraphy	31 (6.86%)	31 (9.39%)	0 (0.00%)
cardiac_uptake	28 (6.19%)	28 (8.48%)	0 (0.00%)
cardiopathy	116 (25.66%)	92 (27.88%)	24 (19.67%)
family_history_of_ATTR_vr	123 (27.21%)	91 (27.58%)	32 (26.23%)
family_history_of_cardiopathy	234 (51.77%)	161 (48.79%)	73 (59.84%)
family_history_of_neuropathy	166 (36.73%)	126 (38.18%)	40 (32.79%)
gastrointestinal_disturbances	140 (30.97%)	107 (32.42%)	33 (27.05%)
lumbar_canal_stenosis	78 (17.26%)	60 (18.18%)	18 (14.75%)
ocular_disorder	88 (19.47%)	78 (23.64%)	10 (8.20%)
positive_biopsy	10 (2.21%)	10 (3.03%)	0 (0.00%)
renal_dysfunction	45 (9.96%)	22 (6.70%)	13 (10.66%)
sensory_neuropathy	283 (62.61%)	234 (70.91%)	49 (40.16%)
sex	242 (53.54%)	188 (56.97%)	54 (44.26%)
unexplained_weight_loss	152 (33.63%)	81 (24.55%)	71 (58.20%)
ttr_mutation	68 (15.0%)	50 (15.2%)	18 (14.8%)

Abbreviations: *CTS* carpal tunnel syndrome, *TTR* transthyretin

**Table 3:** Performance of the evaluated machine learning models on the test set. The table reports accuracy, precision, recall, F1-score, and ROC-AUC for each classifier

Model	Accuracy_score (5% CI, 95% CI)	Precision_score (5% CI, 95% CI)	Recall_score (5% CI, 95% CI)	F1_score (5% CI, 95% CI)	Roc_auc_score (5% CI, 95% CI)
Random Forest Classifier	0.8172(0.7459, 0.8770)	0.4377 (0.2702, 0.6062)	0.8863 (0.7222, 1.0000)	0.5810 (0.4091, 0.7273)	0.8359 (0.7625, 0.8968)
Gradient Boosting Classifier	0.7878 (0.7131, 0.8607)	0.0998 (0.0000, 0.3333)	0.0575 (0.0000, 0.2000)	0.0705 (0.0000, 0.2222)	0.6598 (0.5275, 0.7827)
XGBoost Classifier	0.7775 (0.6967, 0.8443)	0.3961 (0.2449, 0.5455)	1.0000 (1.0000, 1.0000)	0.5632 (0.3934, 0.7059)	0.8851 (0.8219, 0.9390)
DecisionTree Classifier	0.7780 [0.7047, 0.8525)	0.3867 (0.2307, 0.5385)	0.8881 (0.7273, 1.0000)	0.5343 (0.3590, 0.6866)	0.8880 (0.8258, 0.9362)
Logistic Regression Classifier	0.8373 (0.7705, 0.9016)	0.0000 (0.0000, 0.0000)	0.0000 (0.0000, 0.0000)	0.0000 (0.0000, 0.0000)	0.9013 (0.8454, 0.9482)
KNN Classifier	0.7543 (0.6803, 0.8279)	0.1664 (0.0000, 0.3636)	0.1667 (0.0000, 0.3601)	0.1636 (0.0000, 0.3274)	0.6026 (0.4813, 0.7267)

From a clinical perspective, the analysis identified three key predictors with the highest informative power: BCTS, ataxia, and a family history of neuropathy emerged as the three most significant red flags with the highest informative power. Among them, family history of neuropathy and bilateral CTS emerged as the strongest predictors, showing a consistent and robust positive contribution to the outcome. Conversely, while ataxia also appeared as a relevant predictor, its presence was associated with a lower probability of the subject being classified as affected, highlighting a distinct role compared with the other variables.

## Discussion

To the best of our knowledge, this is one of the first studies in which ML has been applied with a dual purpose in the context of ATTRv. First, this approach was specifically designed to identify which red flags are most strongly associated with a positive genetic test for ATTRv, thereby providing a data-driven prioritization of diagnostic indicators. Unlike traditional univariate or regression-based analyses, this methodology leverages the ability of ML algorithms to capture non-linear relationships and complex interactions between variables, allowing for a more comprehensive characterization of the diagnostic relevance of each feature. The ML results provide both computational and clinical insights. From a computational perspective, tree-based machine learning models consistently outperformed the other algorithms evaluated. Notably, the best-performing model demonstrated strong predictive capabilities and high robustness.

In our analysis, BCTS, ataxia and family history of neuropathy emerged as the red flags most strongly associated with a positive genetic test for ATTRv. These findings are consistent with previous reports, in which the same three features were among the most frequently observed clinical indicators in patients with genetically confirmed ATTRv [15–17]. However, in contrast, other studies have reported different features as being more strongly associated with a positive genetic diagnosis, including polyneuropathy, unexplained weight loss, gastrointestinal symptoms, autonomic dysfunction, and cardiomyopathy [12, 18, 19]. Interestingly, previous reports have even suggested that BTCS may be associated with a negative genetic test for ATTRv, highlighting the potential non-specificity of this finding in certain clinical contexts [12]. A previous study applying an ML algorithm to ATTRv screening had shown the predictive role of cardiomyopathy and unexplained weight loss in a population of patients from the south of Italy [12]. These apparent discrepancies may be partly explained by methodological differences across studies. In many previous works, feature selection and association analyses were conducted using conventional statistical approaches, which may fail to capture complex, non-linear interactions among clinical variables. Moreover, in the few studies employing ML techniques, the number of clinical features included in the models was often limited, potentially overlooking subtle but relevant combinations of predictors. Secondly, genetic factors might have influenced the results as the distribution of phenotypes in the South of Italy is quite different from the rest of Italy and Europe, as demonstrated by screening studies [20–23]. Another important factor may be the sample size, very limited in previous studies, with consequent low statistical power and generalizability. Conversely, while ataxia also emerged as a relevant predictor, its presence was associated with a lower probability of a positive genetic diagnosis. This observation may reflect the fact that gait impairment is a late and non-specific manifestation of neuropathic or systemic disease, often present in other acquired neuropathies or cardiac conditions unrelated to ATTRv [24]. In clinical practice, this suggests that while gait disturbance should not be ignored, its isolated presence, particularly in the absence of more specific red flags, may not substantially increase the likelihood of ATTRv and should be interpreted with caution.

Second, beyond descriptive or associative analyses, this model was specifically developed and validated to predict ATTRv diagnosis with greater accuracy than conventional clinical decision-making tools. Over the years, increasingly sophisticated and refined diagnostic algorithms have been proposed [25, 26]; however, none have achieved perfect accuracy, leaving a proportion of patients still undiagnosed or diagnosed late. In this study, the ML model was trained on the Western Sicily sub-cohort and evaluated on the geographically distinct Eastern Sicily counterpart. The Eastern Sicily sub-group, which represents the test set, was withheld from all the model development phases. While this approach goes beyond a purely internal validation, the two sub-cohorts share a similar regional genetic background and referral context, and generalizability to other geographic or clinical settings should therefore be interpreted with caution [27]. By integrating multiple clinical domains and leveraging advanced pattern-recognition methods, the model may represent a promising tool to support the identification of patients who could benefit from confirmatory genetic testing. However, prospective and multi-center validation is required before any clinical implementation.

The comparative analysis of ML models showed that tree-based algorithms consistently outperformed others, maintaining strong performance across various feature setups. This pattern may reflect either the inherent structure of the classification problem or the categorical nature of the predictors used in the dataset. Future studies on independent cohorts will be necessary to determine whether this behavior is specific to the domain or a more general characteristic of the data, and to assess if tree-based algorithms are naturally better suited for this type of problem. From a clinical perspective, three variables stood out as the most informative predictors: family history of neuropathy, ataxia, and bilateral carpal tunnel syndrome. Their strong predictive ability highlights their potential as key clinical markers for disease detection, emphasizing their importance not only statistically but also in diagnosis and patient assessment. The novelty of combining these two goals—finding high-yield diagnostic red flags and developing a reliable predictive model—underscores the translational potential of this research. This dual approach not only enhances understanding of the phenotypic patterns linked to ATTRv but also represents a preliminary framework for the potential future integration of AI into diagnostic workflows, pending prospective validation in independent and geographically diverse cohorts.

## Limitations

It is important to emphasize that our findings derive from a pre-selected cohort of patients referred for suspected ATTRv based on the presence of clinical red flags. Therefore, the identified predictive value of these red flags should not be interpreted in the context of the general population, but rather within a clinically enriched diagnostic setting with a higher pre-test probability of disease. Another limitation of this study is it was conducted at a tertiary referral center for amyloidosis, with a predominantly neurological referral pattern. This may introduce referral bias, as the prevalence and predictive value of red flags may differ in non-specialized or cardiology-based settings, potentially limiting generalizability.

A relevant limitation of this study is the inclusion of variables such as bone scintigraphy, cardiac uptake, and biopsy findings, which may represent confirmatory or late-stage diagnostic features rather than early clinical red flags. Their inclusion reflects the real-world clinical context of this study, in which patients were already referred for suspected ATTRv and may have undergone instrumental investigations before or alongside genetic testing. Generalizability to earlier or non-specialized diagnostic settings should therefore be interpreted with caution. Although the evaluation was conducted on a geographically distinct and separately collected cohort, both subgroups originate from the same regional context and may share similar genetic, clinical, and referral characteristics. Based on this evidence, external validation in cohorts from different geographic and clinical settings remains a necessary next step before clinical implementation, as recommended by TRIPOD + AI [28]. Furthermore, although the Western and Eastern Sicily sub-cohorts differ in size (*N* = 330 and *N* = 122, respectively), the prevalence of TTR mutation was virtually identical across the two groups (15.2% vs. 14.8%). This suggests that the samples’ imbalance did not substantially affect class distribution or model performance.

The genetic landscape of ATTRv in Sicily is characterized by a relatively high prevalence of mutations associated with a predominantly neuropathic phenotype, such as F64L. This may have influenced the distribution and relative importance of red flags identified by the model, with a potential overestimation of neuropathic features (e.g., BCTS, ataxia, and family history of neuropathy) and underrepresentation of cardiologic manifestations. Therefore, model performance and feature importance should be interpreted in the context of this specific genetic and phenotypic background, which may limit generalizability to other populations with different mutational spectra.

This study shows minimal overlap with the previous studies from our group [12]. However, only 12 ATTRv patients of the previous study (12/397, 3%) were included in the present analysis (12/452, 2% of the study sample).

## Data Availability

The data that support the findings of this study are available from the corresponding author upon reasonable request.
